# Interhemispheric visual competition after multisensory reversal of hemianopia

**DOI:** 10.1111/ejn.14554

**Published:** 2019-09-05

**Authors:** Alexander S. Dakos, Ellen M. Walker, Huai Jiang, Barry E. Stein, Benjamin A. Rowland

**Affiliations:** ^1^ Department of Neurobiology and Anatomy Wake Forest University School of Medicine Winston‐Salem NC USA

**Keywords:** cat, competitive stimuli, cortical blindness, superior colliculus, visual rehabilitation

## Abstract

Unilateral lesions of visual cortex have the secondary consequence of suppressing visual circuits in the midbrain superior colliculus (SC), collectively producing blindness in contralesional space (“hemianopia”). Recent studies have demonstrated that SC visual responses and contralesional vision can be reinstated by a non‐invasive multisensory training procedure in which spatiotemporally concordant visual‐auditory pairs are repeatedly presented within the blind hemifield. Despite this recovery of visual responsiveness, the loss of visual cortex was expected to result in permanent deficits in that hemifield, especially when visual events in both hemifields compete for attention and access to the brain's visuomotor circuitry. This was evaluated in the present study in a visual choice paradigm in which the two visual hemifields of recovered cats were simultaneously stimulated with equally valent visual targets. Surprisingly, the expected disparity was not found, and some animals even preferred stimuli presented in the previously blind hemifield. This preference persisted across multiple stimulus intensity levels and there was no indication that animals were less aware of cues in the previously blind hemifield than in its spared counterpart. Furthermore, when auditory cues were combined with visual cues, the enhanced performance they produced on a visual task was no greater in the normal than in the previously blind hemifield. These observations suggest that the multisensory rehabilitation paradigm revealed greater inherent visual information processing potential in the previously blind hemifield than was believed possible given the loss of visual cortex.

## Graphical abstract

Animals with hemianopia can be rehabilitated through the repeated presentation of multisensory stimuli in the affected hemifield. When visual stimuli are presented to these animals competitively, with one in each hemifield, they remain able to detect and in some cases prefer the stimulus in the previously blind hemifield. The addition of auditory components can also provide a behavioral benefit.

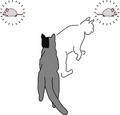

**Abbreviations**
LED: Light‐emitting diode
mcd: millicandelas
SC: superior colliculus

## 1 INTRODUCTION

A common consequence of extensive damage to visual cortex on one side of the brain is a profound blindness in contralesional space (Bolognini, Rasi, Coccia, & Làdavas, 2005; Dundon, Bertini, Làdavas, Sabel, & Gall, 2015; Dundon, Làdavas, Maier, & Bertini, 2015; Frassinetti, Bolognini, Bottari, Bonora, & Làdavas, 2005; Holmes, 1918; Jiang, Stein, & McHaffie, 2009, 2015; Leo, Bolognini, Passamonti, Stein, & Làdavas, 2008; Lomber, Payne, Hilgetag, & Rushmore, 2002; Romano, 2009; Scarlett, 1922). The blindness, or “hemianopia,” is induced because such lesions not only physically damage visual cortex, but also indirectly compromise the functional integrity of other, physically intact, visual processing circuits in the same hemisphere (Jiang et al., 2009). Of principal concern in this context is the midbrain superior colliculus (SC). This structure is primarily involved in detecting and orienting to visual targets (Stein & Meredith, 1993).

In a series of experiments in cat that were inspired by Sprague and colleagues (e.g., Lomber & Payne, 1996; Sherman, 1974, 1977; Sprague, 1966; Sprague & Meikle, 1965; Wallace, Rosenquist, & Sprague, 1989, 1990), the lesion‐induced hemianopia was postulated to be due to the creation of an “imbalance” between excitatory and inhibitory tectopetal inputs. In the absence of counterbalancing excitatory inputs from the lost cortex, SC neurons were suppressed by inhibitory inputs from the opposite side of the brain. Disrupting those inhibitory inputs with strategically placed lesions in the opposite hemisphere, or by cutting their interhemispheric projections, resolved the hemianopia by restoring “balance” to this circuit (Lomber & Payne, 1996; Lomber et al., 2002; Sherman, 1974; Sprague, 1966; Wallace et al., 1989, 1990).

More recent work has shown that hemianopia can be reversed without resorting to invasive strategies. A sensory training program that leverages the multisensory nature of the cat SC has proven to be highly effective in reversing hemianopia. It involves repeatedly presenting a pair of congruent visual‐auditory stimuli in the blind hemifield (Jiang et al., 2015). These findings are consistent with the principles governing SC multisensory integration (e.g., see Meredith & Stein, 1986a; Meredith, Nemitz, & Stein, 1987; Stein & Meredith, 1993) and multisensory plasticity (Yu, Rowland, Xu, & Stein, 2013). The exposure paradigm reinstates the visual responses of multisensory SC neurons that were compromised by the visual cortex lesion, thereby reinstating vision in the blind hemifield (Jiang et al., 2015). It does so despite the normal complement of (inhibitory) inputs from the contralesional hemisphere, presumably by restoring the functional interhemispheric balance.

But, whether this recovery extends to more natural circumstances in which both hemifields contain important visual information is unknown. Often multiple behaviorally relevant external events provide visual stimuli (with or without their natural auditory counterparts) simultaneously in the two hemifields, thereby competing for control of the brain's visuomotor circuitry. The damaged hemisphere would appear to be at a considerable disadvantage under such circumstances, a possibility that has important implications for the multisensory rehabilitative strategies that are used with human patients (Bolognini, Leo, Passamonti, Stein, & Làdavas, 2007; Bolognini et al., 2005; Dundon et al., 2015; Frassinetti et al., 2005; Leo et al., 2008). In fact, even a slight disadvantage would predict complete dominance of equally important attractors in the normal hemifield. The present experiments sought to examine this question directly, and thereby assess the operational effectiveness of this non‐invasive rehabilitative approach. They also probed integration capabilities in the previously blind hemifield and whether the heuristic of inverse effectiveness could explain the overall results. Rehabilitated hemianopic cats, trained to respond to a visual stimulus in either hemifield, were presented with identical (competing) stimuli in homologous regions of the two visual fields. The results of these tests were unexpected: responses to stimuli in the rehabilitated field were robust to competition from the intact field.

## 2 METHODS

### 2.1 Animals

Three adult mongrel cats (1 male and 2 female), 2–6 years of age weighing 3–5 kg were obtained from a USDA‐licensed commercial animal breeding facility (Liberty Research, Inc., Waverly, NY) and are referred to here as F1, F2 and F3 (F1: Female, F2: Male and F3: Female). All procedures were performed in compliance with the 8th Edition of the “Guide for the Care and Use of Laboratory Animals” (National Research Council of the National Academies, 2011) and approved by the Institutional Animal Care and Use Committee at Wake Forest University School of Medicine. Animals were trained and tested in visual and auditory orientation tasks (see below). They were food‐restricted to maintain motivation, but kept within 85% of their free‐feeding weight and fed to satiation in the testing apparatus at the conclusion of each testing day. These animals were used in a previous study (Dakos, Jiang, Stein, & Rowland, 2019).

### 2.2 Rapid sensory assay/screening procedure

A rapid assay was first used to determine the suitability of the animals for these experiments (Jiang et al., 2009, 2015). Animals were gently held by one of the experimenters at the start position, facing the back wall approximately 58 cm away. To establish fixation, the other experimenter stood behind the back wall and presented a small food reward through a hole at the 0° position. That experimenter also ensured that the test did not begin if the animal's eyes deviated from fixation, and either presented a ping‐pong ball at the end of a steel wand (visual stimulus) from behind a black curtain or tapped the ball against the chamber wall while still obscured (auditory stimulus). These stimuli were delivered at randomly selected locations within the central 90° of the visual field (locations selected in 15° increments). All animals responded rapidly to these stimuli, generally approaching them directly to receive a 175 mg food‐pellet (Hill's Science Diet).

### 2.3 Visual orientation training/testing

**Figure 1:**
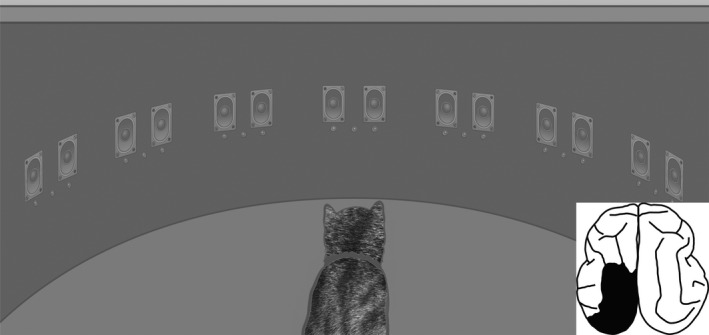
The perimetry apparatus. Arrays of 3 red LEDs and 2 speakers were embedded in the perimetry wall along the horizontal meridian at 15° intervals. Only the middle LED at a given location was used here. During behavioral assessments and training the animal fixated on the central visual stimulus (i.e., the illuminated LED at 0°), then oriented toward, and approached, the visual stimulus appearing at a random location (single‐stimulus) or chose between two visual stimuli appearing simultaneously at −45° and 45° of fixation (choice‐stimulus) with or without a congruent auditory stimulus. The animal was rewarded for approaching any of the LED targets and for remaining at the start position during auditory‐only and catch trials adapted from (Rowland et al., 2007). Inset: Tracing of lesion produced in animal F3. Size and shape of lesion were uniform across all animals

Thereafter, each animal was trained in visual fixation and approach responses in a 90 cm diameter perimetry apparatus equipped with arrays of light‐ emitting diodes (LEDs, Lumex Opto/Components model 67‐1102‐ND) and speakers (Panasonic model 4D02C0) placed at 15° increments from −105° to 105° of central space (Figure 1, see also Gingras, Rowland, & Stein, 2009; Rowland, Stanford, & Stein, 2007). Animals were trained to fixate on the central LED at 0° and, when this fixation stimulus was extinguished, to approach a target LED that became illuminated after a delay of 500 ms at a randomly selected eccentricity. Stimulus intensity was 6 millicandelas (mcd), and stimulus duration was 100 ms. Target locations were restricted to the central 90° of visual space as shown in Figure 1. Interleaved with these stimulus trials were “catch” trials containing no visual stimulus, and maintenance of fixation was rewarded. No auditory cues were used. Training was complete when an animal achieved criterion performance of >85% correct. In order to limit any experimenter‐specific biases, each animal was trained and tested by multiple experimenters. Additionally, each experimenter used headphones to block auditory cues and avoided observing the LED display until well after each animal's response was scored; thus, stimulus location was unknown to the experimenter until after an animal made its choice. No difference was seen in animal performance across animal gender or experimenter.

### 2.4 Induction of hemianopia

Prior studies have indicated no significant differences between left and right side lesions in this preparation; thus, for consistency, all hemianopia‐inducing lesions were made on the left (Jiang et al., 2015). Briefly, each animal was anesthetized with acepromazine/buprenorphine (0.02–0.05/0.005–0.01 mg/kg, IM) and sodium pentobarbital (22–30 mg/kg, IV), and its head fixed in a stereotaxic frame. Next, a craniotomy was performed to expose visual cortex. All contiguous areas of visual cortex were targeted for aspiration as in (Jiang et al., 2015). This included most of the posterior lateral and suprasylvian gyrus, rostral portions of the posterior ectosylvian sulcus (sparing the anterior region) and the cortical area above the splenial sulcus posterior to the cruciate gyrus (Figure 1, inset). The size and completeness of the lesion eliminates any concern over possible spared visual tissue. The lesion site was packed with Gelfoam, the bone flap was replaced and sutures were used to seal the scalp incision. Antibiotics (cefazolin, 25 mg/kg, IM), analgesics (buprenorphine, .01 mg/kg, IM) and saline (60 ml, SQ) were administered after the procedure. Ipsiversive circling was noted immediately following recovery from the surgery, but decreased rapidly thereafter. However, a profound contralateral blindness was apparent, and this persisted unchanged throughout the 2.5–3‐ month observation period needed to ensure the stability of the visual defect (Jiang et al., 2015; Sprague, 1966; Wallace et al., 1990).

### 2.5 Multisensory rehabilitation

After determining that the visual defect was stable, animals underwent a daily multisensory training procedure that was designed to reestablish vision in the blind hemifield (Jiang et al., 2015). The apparatus and basic behavioral paradigm (i.e., fixation, orientation and reward) for this procedure were similar to those described in “Visual Orientation Training/Testing” above. However, here the stimulus set consisted of visual alone trials (“single stimulus” trials involving no choice) in the normal hemifield (*N* = 20), and multisensory (visual‐auditory) rehabilitation trials (*N* = 50) in the blind hemifield. In these multisensory rehabilitation trials, the visual stimulus was at 45° coupled with a concordant 100 ms broadband auditory stimulus. Catch trials (no stimulus) were interleaved. Each day, at the completion of the training trials, individual visual stimuli were presented at all eccentricities to determine if, when, and where visual responses could be elicited in the previously blind hemifield. After 7 weeks, all animals responded briskly to visual stimuli at all tested eccentricities in the previously blind hemifield, confirming the effectiveness of the rehabilitation procedure in reestablishing vision.

### 2.6 Visual choice tests

After rehabilitation, competitive tests were begun to evaluate the relative efficacy/robustness of the visual processing in that hemifield. In these “visual‐choice” trials (*N* = 200/animal) identical visual stimuli (100 ms LED flash, as in other tests) were presented simultaneously at −45° and 45° (i.e., in opposing hemifields). The animal was rewarded equally for responding to either stimulus on a given trial so that each of these valid “targets” competed for a response. These trials were interleaved with catch trials and individual presentations of visual or auditory stimuli at −45° and 45°. Animals were trained not to respond to auditory stimuli when presented alone and were rewarded for not approaching them (i.e., a NO–GO response).

In choice trials animals showed clear preferences for stimuli on a given side of space, and once the magnitude of this preference was established, the intensity of the visual stimulus on the “preferred” side was systematically lowered to eliminate that preference and render the two stimuli equally effective. This was done to ensure that the preference was tied to the stimulus and not the side of space. Thereafter, the intensity of the visual stimulus on the previously “non‐preferred” side was lowered so that the two stimuli were now of equal intensity at the new lower level. This was done to see whether the previous preference was reestablished to ensure that preferences were stable at multiple stimulus levels.

### 2.7 Multisensory enhancement tests

**Figure 2:**
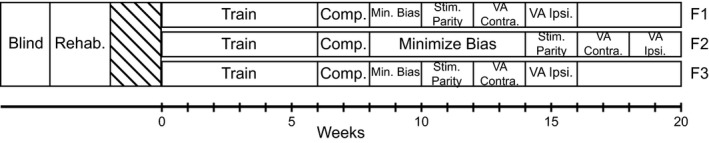
Training Timeline. Each animal had been rehabilitated from hemianopia. Following a break period, each animal was trained using competitive visual stimuli. Animals were then tested with competitive visual and multisensory stimuli

To evaluate the ability of these animals to use visual and auditory information synergistically in the previously blind hemifield, visual‐auditory stimulus pairs were added to the visual choice paradigm. Traditional evaluations of multisensory integration have demonstrated that the addition of a spatiotemporally congruent auditory stimulus (even one trained to elicit a NO–GO response) greatly enhances the detection and localization of a weakly effective visual stimulus (Gingras et al., 2009; Jiang, Jiang, & Stein, 2002; Rowland et al., 2007; Stein, Meredith, Huneycutt, & McDade, 1989). Here, multisensory tests were conducted in which a weak auditory stimulus consisting of a 100 ms broadband noise burst was presented congruently with either the −45° or 45° visual stimulus in choice trials. Visual stimulus intensities in these trials were tested in two configurations: when adjusted to eliminate the animal's native preference/bias, and at the equal but lower intensity level described above. Single‐visual, visual‐choice, single‐auditory, visual‐auditory and catch trials were all tested in an interleaved fashion with equal incidence. A timeline is provided as a summary of each animal's training (Figure 2).

### 2.8 Paw preference

To examine whether preferences for visual stimuli on one side or the other were related to paw preference (“handedness” in cats) each animal's paw preference was also evaluated. The forelimb used most often in reaching through the gratings of its home cage or carrier to obtain an offered food reward was tested on 30 trials. Animals were said to prefer a particular paw if they reached with it on at least 2/3 of the trials.

### 2.9 Data analysis

Orientation response data were pooled across trials and days for each animal and test condition. Responses to single stimuli were classified as accurate/inaccurate and the percent of accurate responses calculated. Response preferences in choice tests were similarly quantified as the percentage of responses to each side. Multisensory enhancement was quantified as the raw difference between the response preferences on multisensory trials versus the matching preferences when only visual choice stimuli were presented. Comparisons of accuracy and preference between stimulus conditions were conducted with Fisher's exact test. Evaluations of preference on choice trials used binomial tests. Regression was used to identify relationships between multisensory enhancement and visual‐only choice performance in order to determine whether an inverse effectiveness trend existed. Enhancement is defined as raw difference in orientation preference (i.e., VA–V) for tested conditions on each side of the visual field. Pooled *R* ^2^ and *p*‐values for these relationships were calculated for the group. Alpha was 0.05. Data were analyzed in MATLAB (v.9.1; MathWorks).

## 3 RESULTS

The primary finding was that the multisensory exposure procedure reinstated visual detection, localization and orientation performance in the previously blind hemifield, and did so at a level that rivaled that in the normal hemifield and in normal animals. There was no evidence of a competitive disadvantage in the previously blind hemifield in responding to visual events or in the use of auditory cues to enhance performance on these tests.

### 3.1 The results of single‐stimulus tests

**Figure 3:**
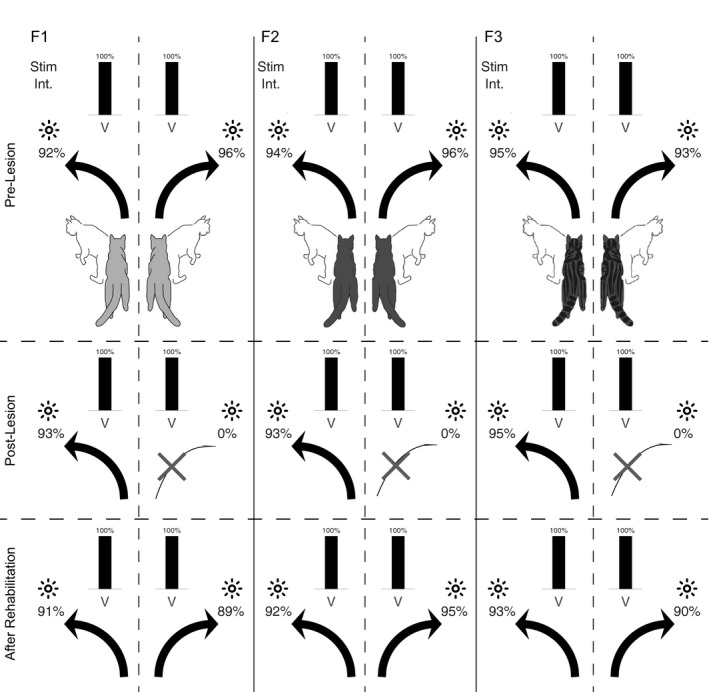
Results for single‐stimulus tests. Illustrated is the accuracy with which each animal (F1, F2 and F3) responded to the brightest visual (LED, black bar) stimulus when presented individually on either side of space (−45° and 45°). Numbers indicate performance accuracy as percent correct. Top Row: Visual performance on single‐stimulus trials before the visual cortex lesion. Middle Row: Performance after the visual cortex lesion. Note the loss of visual responses in the contralesional (right) hemifield. Bottom Row: Performance after multisensory rehabilitative training. Note the return of vision in the previously blind hemifield, and that performance rivaled that in the normal hemifield (1,000–1,100 trials/animal/stimulus). There were no interhemispheric differences in accuracy observed for either modality (F1:91%/89% visual, *p* = .48; F1:98%/98% auditory, *p* = .57; F2:92%/95% visual, *p* = .19; F2:96%/94% auditory *p* = .35 and F3:93%/90% visual, *p* = .26; F3:98%/99% auditory, *p* = .41)

As shown in Figure 3, near‐equivalent mean accuracy scores were achieved by each animal in orienting to −45° and 45° visual targets before visual cortex was ablated and after multisensory rehabilitation training reinstated vision in the blind hemifield. None of the animals had difficulty in maintaining fixation, none exhibited any obvious abnormal visuomotor behaviors, and all responded briskly and accurately when presented with a single‐visual stimulus at its brightest level in both hemifields. It appeared that the multisensory training had restored visuomotor performance in this context to normal levels, as no animal showed significant hemispheric performance differences. An equivalent number of single‐stimulus auditory trials were interleaved within all competitive stimulus conditions (not shown). Similarly, all animals showed near‐perfect performance (94%–99% accuracy) in their NO–GO tests with auditory stimuli in either hemifield. In short, there were no interhemispheric differences in accuracy observed for either modality (F1:91%/89% visual, *p* = .48; F1:98%/98% auditory, *p* = .57 and F2:92%/95% visual, *p* = .19; F2:96%/94% auditory *p* = .35; F3:93%/90% visual and *p* = .26; F3:98%/99% auditory, *p* = .41).

### 3.2 Visual choice tests

**Figure 4:**
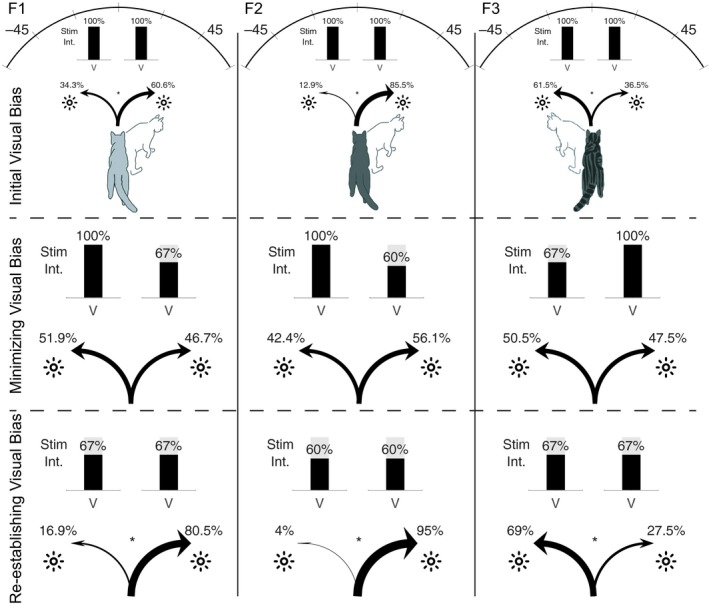
Visual choice tests. Top Row: In the initial choice tests (200–240 trials/animal), an LED was illuminated at its highest level (100%, ~6 mcd) simultaneously in the two hemifields. Animals F1 and F2 showed a 26% and 73% preference for the contralesional (previously blind) hemifield (F1: 60.6%, *p* = .00068 and F2: 85.5%, *p* = 4.5e−26), whereas animal F3 showed a 25% preference for the ipsilesional (normal) hemifield (F3: 61.5%, *p* = 2.8e−4). Middle Row: Reducing stimulus intensity in the preferred hemifield to 60%–70% of maximum minimized the bias in all animals (animal F1 now showed a slight preference for the ipsilesional hemifield), (preference change: F1: 60.6% to 46.7%, *p* = .0059 [6 to 4 mcd]; F2: 85.5% to 56.1%, *p* = 6.8e−7 [6 to 3.6 mcd] and F3: 61.5% to 50.5%, *p* = .017 [6 to 4 mcd]). Bottom Row: Equilibrating stimuli in the two hemifields at that lower value reinstated the initial hemispheric bias, revealing an even greater magnitude of bias at the lower intensity. F1 and F2 again preferred the previously blind side (F1: 80.5%, *p* = 6.1e−7, F3: 95% and *p* = 5.4e−12) and animal F3 the normal side (69%, *p* = 4.8e−5)

Interleaved with post‐rehabilitation single‐stimulus tests were “visual choice” tests in which identical visual stimuli were simultaneously presented on the left (−45°) and right (45°). Animals were rewarded for a response to either stimulus so that the stimuli were effectively competing for an overt orientation response. Here interhemispheric differences were observed as shown in Figure 4. But, contrary to expectation, there was no apparent general advantage of the intact hemisphere. Only one animal exhibited a bias toward stimuli in the ipsilesional (F3: 61.5%, *p* = 2.8e−4). The other two animals preferred stimuli in the previously blind hemifield (F1: 60.6%, *p* = .00068 and F2: 85.5%, *p* = 4.5e−26). The mechanisms underlying the side preference were not explored here; however, it was roughly consistent with paw preference: although animal F2 (right stimulus‐preferring) had no discernable paw preference, F1 preferred the right stimulus and also its right paw while F3 preferred both the left stimulus and its left paw. Similar consistency between handedness and laterality preferences in auditory and sometimes visual tasks have been observed in humans (McLaughlin, Dean, & Stanley, 1983; Scharine & McBeath, 2002). Note that all visual stimuli were at their highest level, and the perceptibility of the individually‐presented stimuli was near 100% in both hemifields.

The absence of a competitive advantage of the intact hemisphere prompted additional tests to determine whether the preference would appear when the stimuli were more difficult to detect. The intensity of the stimulus on the preferred side was systematically manipulated in each animal to determine what level of reduction was necessary to minimize the initial preference and equilibrate performance to stimuli in the two hemifields.

### 3.3 Equilibrating hemispheric preference

Reducing the intensity of the visual stimulus on the “preferred” side to between 3.5 and 4 mcd equilibrated the initial hemispheric preferences in each animal (preference change: F1: 60.6% to 46.7%, *p* = .0059 [6 to 4 mcd]; F2: 85.5% to 56.1%, *p* = 6.8e−7 [6 to 3.6 mcd] and F3: 61.5% to 50.5%, *p* = .017 [6 to 4 mcd]). This change was similar or identical across animals regardless of which hemisphere was initially preferred.

After testing at these levels, the stimulus intensity on the previously “non‐preferred” side was lowered so that the two stimuli intensities were again identical. As expected, this (Figure 4, bottom row) reinstated the animals’ initial side preferences (Figure 4, top row); that is, at the lower (matched) intensity levels, animals F1 and F2 again preferred the previously blind side (F1: 80.5%, *p* = 6.1e−7 and F3: 95%, *p* = 5.4e−12) and animal F3 the normal side (69%, *p* = 4.8e−5). In fact, the preference at the lower intensity level was stronger than observed at the higher intensity level in one animal (F1: *p* = 4.1e−4; F2: *p* = .25 and F3: *p* = .70). In sum, animals’ stimulus/side preferences were not predicted by the side of the lesion and persisted across multiple stimulus intensity levels.

### 3.4 Multisensory enhancement

Spatiotemporally congruent auditory stimuli typically enhance the physiological and perceptual salience of visual stimuli in normal animals, increasing the speed and accuracy with which they can be detected and localized (Gingras et al., 2009; Goldring, Dorris, Corneil, Ballantyne, & Munoz, 1996; Jiang et al., 2002, 2015; Meredith & Stein, 1986a; Meredith et al., 1987; Rowland et al., 2007; Stanford, Quessy, & Stein, 2005; Stein et al., 1989). These enhancements are proportionately greatest when the visual stimuli are weakly effective or ambiguous (the “principle of inverse effectiveness”) (Gingras et al., 2009; Meredith & Stein, 1986b; Stanford et al., 2005; Stein & Meredith, 1993). To determine whether the reinstated visual processing could be augmented by auditory input as in normal animals, multisensory enhancement capabilities were assessed and compared in both hemifields within the choice paradigm.

**Figure 5:**
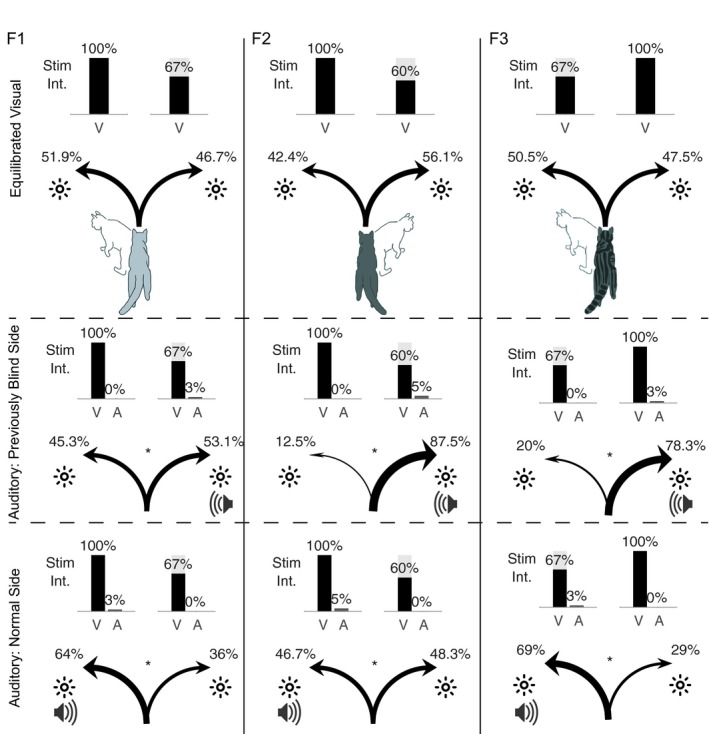
Multisensory enhancement. Top Row: Performance levels after visual stimulus intensities were adjusted to mitigate/eliminate preference in the choice paradigm (same as Figure 3, middle row) (*N* = 200 trials/animal). Middle Row: Coupling the auditory and visual stimuli in the previously blind (right) hemifield biased preference toward that cross‐modal stimulus (F1: 46.7% to 53.1%, *p* = .035; F2: 56.1% to 87.5%, *p* = 7.4e−8; F3: 47.5% to 78.3% and *p* = 9.5e−7). Bottom Row: A shift in preference (from top row) to the cross‐modal stimulus was also produced in the normal (left) hemifield (F1: 51.9% to 64%, *p* = .013; F2: 42.4% to 46.7%, *p* = .041 and F3: 50.5% to 69%, *p* = .0014). Note that the preference was no greater than in the previously blind hemifield (*p* = .59)

After re‐adjusting visual intensities to mitigate/eliminate the initial choice preferences (Figure 5, top row), a low intensity 100 ms duration broadband auditory stimulus was simultaneously presented in spatiotemporal concordance with one of the visual stimuli in the visual choice paradigm. When presented with the visual stimulus in the normal hemifield, the auditory stimulus produced a significant increase in animals’ preference for it (F1: 51.9% to 64%, *p* = .013; F2: 42.4% to 46.7%, *p* = .041 and F3: 50.5% to 69%, *p* = .0014) (Figure 5, middle row). There was no apparent relationship between the magnitude of this enhancement and whether the animal preferred that visual stimulus (side) when visual intensities were equal: equal enhancements were observed for right‐preferring F1 and left‐preferring F3 (*p* = .71).

The auditory stimulus also enhanced the animals’ preference for the visual stimulus in the previously blind hemifield when presented congruently with it (F1: 46.7% to 53.1%, *p* = .035; F2: 56.1% to 87.5%, *p* = 7.4e−8 and F3: 47.5% to 78.3%, *p* = 9.5e−7). Enhancement magnitudes were not consistently greater in the normal or previously blind hemifield across animals (*p* = .59). And as above, there was no consistent relationship between the native side preference and the magnitude of this enhancement: equal enhancements were observed for right‐preferring F2 and left‐preferring F3 (*p* = .15).

**Figure 6:**
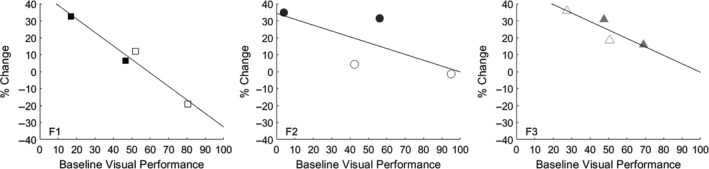
Inverse Effectiveness. Each plot represents data from one animal (F1–F3, left–right). Filled symbols represent stimulus presentations in the normal hemifield, and open symbols represent presentations in the previously blind hemifield. The horizontal axis represents each animal's baseline performance in orienting to a visual stimulus in the presence of a competing visual stimulus. The vertical axis represents the percentage change in performance after coupling a weakly effective auditory stimulus with the visual stimulus. As predicted by the “principle of inverse effectiveness,” each animal displayed decreased multisensory enhancement when the auditory stimulus was combined with a visual stimulus of greater preference. Solid lines are least‐squares regression fits. Inverse trend is significant (*R* ^2^ value = .50, *p* = .0067)

However, there was considerable variance in the levels of multisensory enhancement observed across animals and sides of space (Figure 5). To better appreciate the sources of this variance, animals were also tested with visual‐auditory pairs in the choice paradigm when visual stimulus intensities were equal on both sides of space (3–4 mcd). After pooling all multisensory tests separately for each animal, it was apparent that a major source of variance in multisensory enhancement was the level of preference for the visual stimulus to which the auditory was coupled. In short, visual stimuli that were less preferred were more enhanced when combined with the auditory stimulus, while highly preferred visual stimuli were less enhanced (Figure 6). This is evidence of the “principle of inverse effectiveness” consistently observed in studies of multisensory enhancement (Meredith & Stein, 1986b; Stein & Meredith, 1993; Stein, Stanford, Ramachandran, Perrault, & Rowland, 2009). Pooled regression was significant (adjusted *R* ^2^ value = .50, *p* = .0067).

## 4 DISCUSSION

### 4.1 Restoring vision in the blind hemifield

The hemianopic animals rehabilitated by multisensory training showed robust contralesional visual detection and localization capabilities, yet did so with a much diminished visual circuitry. Nevertheless, and despite the absence of visual cortex, there was no evidence that the damaged hemisphere suffered any disadvantage when competing with its undamaged counterpart in choosing between identical visual stimuli. The animals detected, located and approached the visual stimulus in the previously blind hemifield as well as in the opposite (normal) hemifield. In fact, in competitive choice tests, 2/3 animals preferred visual stimuli in that hemifield over those in the normal hemifield when matched in intensity. A significant asymmetry in stimulus intensity was required to eliminate that preference. There was also no evidence that the lesion had compromised the multisensory processing capabilities of the damaged hemisphere: interactions among auditory‐visual stimuli were as effective in enhancing choice in the previously blind hemifield as they were in the normal hemifield.

For some time now, it has been known that the lesion‐induced hemianopia occurs because the functional consequences of the lesion extend well beyond what would be expected of damage restricted to the lesion site. These lesions also functionally inactivate the visual processing of neurons in the midbrain SC, rendering them incapable of supporting contralesional vision and effectively blinding the animal in that hemifield (Jiang et al., 2015). Presumably, the lesion does so by disrupting the interhemispheric “balance” of excitation and inhibition that exists between the visual cortices and SC on one side of the brain and their counterparts on the other side (Sprague, 1966). Lesions of anterior ectosylvian sulcus (AES) reinstate blindness in rehabilitated animals (Jiang et al., 2015). When a potent source of excitation to the ipsilesional SC is removed, its visual activity becomes suppressed by inhibitory inputs from the basal ganglia and the contralateral SC that traverse the intercollicular commissure. This conclusion is based on observations that secondary lesions in the opposite hemisphere that eliminate this inhibitory input establish a new balance (along with new visual disruptions), thereby restoring vision to the blinded hemifield. This phenomenon is known as the “Sprague Effect” (Lomber et al., 2002; Sherman, 1974, 1977; Sprague, 1966; Wallace et al., 1990;).

In the present study, highly effective visual processing was established for contralesional space without any such physical interruption of interhemispheric pathways. The absence of a competitive disadvantage in the damaged hemisphere suggests that, after multisensory rehabilitation, the presence of visual cortex provides no obvious advantage in visual detection/localization/choice tests—at least not in response to stimuli such as those used in these experiments. This was surprising and demonstrates that, despite the obvious heuristic value in the concept of interhemispheric “balance”, it does not fully capture the dynamics among the component structures or their individual functional potential. It is also surprising given the common perspective that loss of visual cortex should render the animal unaware of visual stimuli in the contralesional hemifield: these animals showed no evidence of diminished awareness. Additionally, ceiling performance in orienting to a single stimulus in either hemifield suggests a lack of a spatial location bias.

Equally surprising in this context is the finding that some visual pattern discrimination capabilities are present in the compromised hemisphere after multisensory rehabilitative training (Jiang et al., 2015). This capability is commonly regarded as cortically‐based, although there is some evidence that the midbrain can also participate in this function (Berkley & Sprague, 1979; Doty, 1971; Schneider, 1969; Sprague, Berkley, & Hughes, 1979). The present experiments also revealed that the rehabilitated hemisphere has the ability to detect stationary flashed stimuli; a capability that appears to be beyond the visual repertoire of animals whose hemianopia was reversed by a lesion mitigating interhemispheric inhibition in the Sprague Effect (Wallace et al., 1989, 1990).

Although the underlying neural mechanisms by which multisensory rehabilitation operates are poorly understood, a key factor is thought to be reinstating the visual activity in the multisensory layers of the SC that were silenced by the lesion (Jiang et al., 2015). Presumably, the repeated bouts of multisensory stimulation during rehabilitative training change the visual sensitivity of these SC neurons (e.g., by lowering their activation thresholds). Spatiotemporally concordant auditory‐visual stimuli such as those used in this rehabilitative training have been shown to render the visual responses of multisensory SC neurons more robust, presumably by shifting their response function toward lower intensities (Yu et al., 2013). It is possible that such a mechanism underlies the changes in multisensory SC neurons that once again render them capable of supporting contralateral visual behavior (see also Jiang et al., 2015).

### 4.2 Multisensory enhancement in the previously blind hemifield

Auditory stimuli enhance the responses of the SC neurons to visual stimuli via multisensory integration (Gingras et al., 2009; Jiang et al., 2002; Meredith & Stein, 1986b; Stein & Meredith, 1993), and similar enhancements have been previously observed in SC‐mediated detection and localization of auditory‐visual stimuli in normal animals (Gingras et al., 2009; Jiang et al., 2002; Meredith & Stein, 1986b; Rowland et al., 2007; Stein & Meredith, 1993; Stein et al., 1989). They also induce a strong bias in visual choice (Onat, Libertus, & König, 2007; Quigley, Onat, Harding, Cooke, & König, 2008), as observed here. Also, as in studies with neurotypic adults, associating the auditory stimulus with a NO–GO response did not preclude its ability to enhance orientation responses to a visual target stimulus (Rowland et al., 2007; Stein et al., 1989). This is revealing, because the auditory and visual stimuli were linked to two different motor plans, and the behavioral enhancement produced by their combination is more consistent with a sensory interaction rather than a race between redundant motor plans (Miller, 1982).

It is important to note that the visual stimuli used here were clearly detectable. Thus, despite the constraint of multisensory integration by the “principle of inverse effectiveness” (Meredith & Stein, 1986b; Stein et al., 2009), the proportionate choice enhancements induced by cross‐modal stimuli in the previously blind hemifield were still substantial (up to 31%) and appeared to be no less robust than those observed in the normal counterpart. The results also indicate that “early” interactions in primary visual cortex (Foxe & Schroeder, 2005; Ghazanfar & Schroeder, 2006; Schroeder & Foxe, 2005;) are not crucial drivers of multisensory enhancements in the response preference observed here. They are also consistent with the idea that multisensory integration plays a primary role in this rehabilitative phenomenon (Jiang et al., 2015), and underscore the fact that significant multisensory benefits are not restricted to any specific behavioral paradigm or stimulus feature (e.g., near‐threshold intensities, cf. [Fetsch, Pouget, DeAngelis, & Angelaki, 2012; ]). This makes sense in the context of the prevailing heuristic in which animals consistently make use of all the information made available by their different senses in making behavioral decisions, whether they are fulfilling a specific task instruction (e.g., detecting a stimulus) or simply selecting a target among alternatives with equal value (Anastasio, Patton, & Belkacem‐Boussaid, 2000; Ernst & Banks, 2002; Knill & Pouget, 2004; Körding & Wolpert, 2004; Ma, Beck, Latham, & Pouget, 2006; Rowland et al., 2007).

Lastly, a caveat should be emphasized. Despite the impressive visual performance of the previously hemianopic animals on these tests, the loss of all contiguous areas of visual cortex surely must have produced significant visual processing deficits. Presumably, these would become apparent in tests requiring more complex form identification and finer levels of visual acuity, tests that were not conducted here. Yet, the fact that the rehabilitative paradigm allows the animals to orient and fixate on a visual target may minimize even these deficits by bringing the target onto the central retina and engaging the intact hemisphere in their evaluation. By eliminating the most profound visual defect (i.e., complete blindness), rehabilitation opens the door to new adaptive strategies that can best exploit the capabilities of the surviving visual circuits.

## CONFLICT OF INTEREST

The authors declare no competing interests.

## AUTHOR CONTRIBUTIONS

Dr. Alexander S. Dakos and Dr. Ellen M. Walker contributed equally. Dr. Alexander S. Dakos and Dr. Ellen M. Walker collected and analyzed the data and prepared the manuscript. Dr. Huai Jiang assisted in data collection and surgical procedures. Drs. Barry E. Stein and Benjamin A. Rowland assisted in planning the experiments and preparing the manuscript.

## Data Availability

Primary data are available from the authors on request.
